# A dataset on energy efficiency grade of white goods in mainland China at regional and household levels

**DOI:** 10.1038/s41597-023-02358-x

**Published:** 2023-07-12

**Authors:** Zonghan Li, Chunyan Wang, Yi Liu

**Affiliations:** School of Environment, Tsinghua University, Beijing, China

**Keywords:** Sustainability, Energy and behaviour

## Abstract

To improve energy-saving management, the energy efficiency grade (EEG) was introduced by the Chinese government in the 2000s and mainly implemented for white goods (WGs) in early stages. However, due to the lack of actual statistics, how effective the promotion of high EEG WGs has been in China is still not clear. The **C**hina **E**nergy **E**fficiency **G**rade (CEEG) of WGs dataset described here comprises (i) EEG-related data on 5 kinds of WGs at the regional (national, provincial) and household levels in China and (ii) predictions of future average EEG trends. By web crawling, retrieving and processing in SQL, the average EEG data weighted by sales in 30 provinces in mainland China from 2012 to 2019 are provided. Household WG survey data, including household information and average EEG, were collected by distributing questionnaires to 1327 households in Beijing, China. The CEEG dataset will facilitate the advancement of research on household energy consumption, household appliance consumer choice, and the assessment of energy efficiency-related policies.

## Background & Summary

White goods (WGs), including washing machines, room air conditioners, and water heaters, are large, important and popular household appliances (HAs)^1^ that account for approximately 40% of noncooking household electricity consumption^2,3^. To improve energy efficiency and energy-saving management, energy efficiency labels (EELs) for WGs were gradually introduced in the 2000s^4^. EELs provide information on the energy efficiency grade (EEG), an integer between 1 (high level, more energy-efficient) and 5 (low level, less energy-efficient), and its related parameters (RPs). After more than a decade of development, both WG EEG standards and energy-saving technologies have been improved^5^. However, how effective the promotion of WGs with high EEGs has been in China is still not clear. It is vital for policy-makers to have a complete view of the EEGs of products sold in the market and to evaluate and adjust the implemented EEG policies and standards.

To the best of our knowledge, there are few statistics that can be implemented to solve the EEG promotion evaluation problem. Scholars have attempted to estimate the energy-saving ability of WGs from a regional perspective. In some studies, sales data from certain online shopping platforms have been introduced^6,7^. However, due to limited actual data, studies are often limited to one or two types of appliances (mostly refrigerators^6–8^), and the time span of the data is relatively short (mostly cross-sectional data). Hence, it may be difficult to accurately understand the overall trends and situations.

In other studies, the aim has been to explore promotion effectiveness by utilizing consumer behavior decision theories. For WGs, purchase behaviors are more heterogeneous^9,10^ among households or individuals. Thus, numerous factors may influence WG purchase behaviors and, consequently, the market share of high EEG WGs. This possibility has increased the need for related questionnaire surveys^11,12^ or simulation experiment^13^ data. However, these approaches are either costly or regionally limited and are difficult to apply in other regions.

In addition to analyzing historical developments in the market share of high EEG WGs, it is vital to forecast future trends by using regional and household statistics and methods. For instance, traditional econometric models^14,15^ and machine learning (ML) algorithms^16,17^ have been widely used in research with similar data sizes. These types of studies allow the current EEG policies and standards to be assessed and can play an important role in effectively and efficiently promoting energy-efficient WGs.

Within these contexts, the **C**hina **E**nergy **E**fficiency **G**rade (CEEG) of WGs dataset is developed in this study. The dataset provides average EEG data from both the regional and household perspectives. It contains 5 kinds of WGs, including impeller washing machines (IWMs), drum washing machines (DWMs), electrical water heaters (EWHs), room air conditioners (ACs), and variable-speed room air conditioners (VACs), which account for 78% of WG-related household electricity consumption in China^3^. The regional part contains sales information for the 5 kinds of WGs and the average EEG weighted by sales data for 30 provinces in mainland China (except for Tibet) from 2012 to 2019. The household part comprises household socioeconomic, demographic, living and EEG-related information on 1327 households in Beijing, China, in 2019 and 2021. Due to the tedious process of raw data acquisition and processing, we also establish models to predict the annual average EEG of the purchased WGs based on socioeconomic information at different scales and provide the prediction results.

In summary, the aims of this study are as follows:

- To collect, process, and provide data related to the EEGs of WGs at the regional (including national and provincial) level from 2012 to 2019 and at the household level in 2019 and 2021.
- To predict EEGs through socioeconomic, demographic and living factors and compare the variables and methods for various prediction models at the regional and household levels.

By providing EEG-related data and prediction models at different spatial scales, the CEEG dataset will facilitate the advancement of research on household energy consumption, HA consumer choice, and the assessment of EEL-related policies.

## Methods

**Fig. 1:**
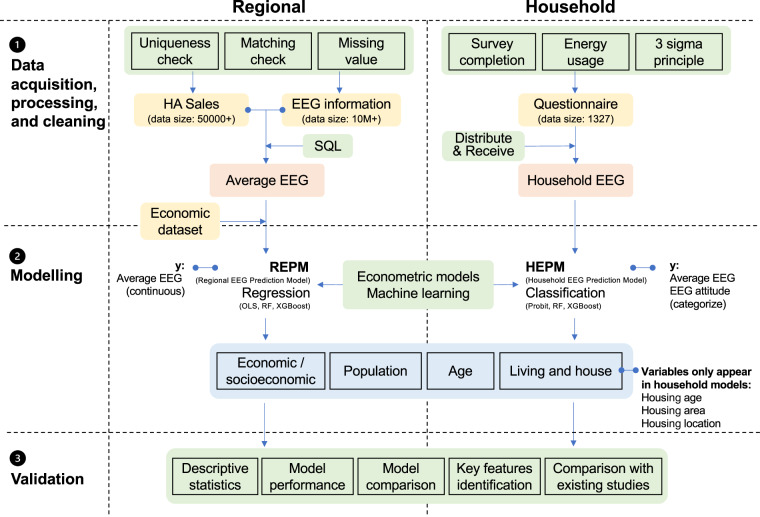
Construction flows of the dataset.

A schematic of the process of generating the CEEG dataset is illustrated in Fig. 1. The research framework consists of three modules: data acquisition, processing and cleaning; modeling; and validation.

- *Data acquisition, processing and cleaning*: We first acquired EEG information, WG sales data, economic/socioeconomic data and household WG survey data by retrieving, web crawling and surveying (detailed in Table 1). Then, we cleaned the EEG information and WG sales data by conducting uniqueness and matching checks, and we cleaned the household WG survey data by applying a survey completion check, energy usage check and 3-sigma principle (detailed in the “Data cleaning” section). Additionally, we attempted to fill in missing values (mainly for EEG information) through manual retrieval (detailed in the “Data cleaning” section). Finally, we used the “query” and “category summary” methods in SQL to fuse and process the EEG information and WG sales data and to calculate average EEG weighted by sales.

**Table 1:** Datasets used to establish the EEG dataset and develop the data-driven models.

Spatial	Data	Source	Acquisition method	Note
National	EEG information	China Energy Efficiency Label Website (https://www.energylabel.com.cn/index.htm)	Web crawler	Before 2019 (For different WG models)
National	EEG information (to complete missing values)	JD.com (https://www.jd.com) Zhongguancun Online (http://www.zol.com.cn)	Retrieval	Before 2019 (For different WG models)
National/Provincial	WG sales	Beijing All View Cloud Data Technology Co., Ltd. (https://www.avc-mr.com/)	Purchase	2012–2019, monthly
National/Provincial	Economic/Socioeconomic data	Wind (https://www.wind.com.cn/NewSite/edb.html)	Retrieval	2012–2019, monthly
Household	Household WG survey	Self-designed-and-distributed questionnaire	Survey	2019 and 2021, yearly

Notes: To complete the missing values in the EEG information dataset acquired by the web crawler, some values were retrieved from online shopping websites. The detailed methods are stated in the “Data cleaning” section below.
- *Modeling*: We deployed traditional econometric methods and ML algorithms to establish a regional average EEG prediction model (REPM) for each kind of WG and a household average EEG prediction model (HEPM) for the average EEG and EEG attitude of the head of household (HH). As the regional average EEG data are continuous and the household data are categorical, regression and classification were employed for the REPM and HEPM, respectively.
- *Validation*: We evaluated and compared the models based on performance indicators (e.g., R^2^), identified key features in the ML algorithms by calculating the importance of the features, and compared our findings with existing studies to validate the credibility of the dataset.

### Data acquisition and processing

To compile the EEG dataset, we used 4 data sources (detailed in Table 1**)**. At the regional level, we integrated EEG information and WG sales datasets. At the household level, we distributed the household WG survey questionnaire to collect household socioeconomic, demographic, living and EEG-related information. To establish the REPM, we also retrieved economic/socioeconomic data (Table 1), including national and provincial macroeconomic and demographic factors.

#### Regional data

The ***EEG information*** dataset contains the EEG and energy efficiency-RPs for each model (a particular type of machine^18^) of each kind of WG. This dataset is used to describe the performance of different WG models in terms of energy savings. According to relevant regulations and EEG standards, manufacturers are required to report the EEG information and energy efficiency-RPs of a HA on the China Energy Efficiency Label Website (https://www.energylabel.com.cn/index.htm) before it is available in the market, and all data on the website are open access to the public. A web crawler was employed to obtain the EEG information of all models of the 5 kinds of WGs included on the website mentioned above from the implementation year of the corresponding EEG standards of the HAs to 2019. For instance, the first edition of the EEG standard for EWHs was implemented in 2008. Thus, the dataset contains EEG information on every EWH model from 2008 to 2019.

**Fig. 2:**
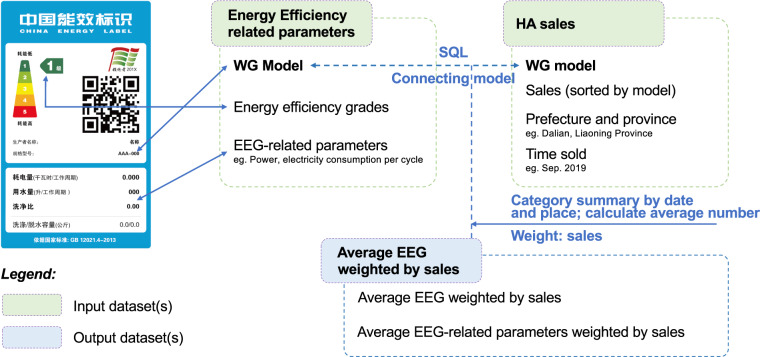
Workflow of the data processing for the **average EEG weighted by sales**.

The ***WG sales*** dataset contains the monthly offline sales amount for each WG model in each prefecture in China from 2012 to 2019 and is employed (i) to analyze the market performance of each kind of WG and (ii) to serve as intermediate values for the average EEG calculations. The dataset was obtained from Beijing All View Cloud Data Technology Co., Ltd. (https://www.avc-mr.com/). SQL was utilized to calculate the **average EEG weighted by sales**, which reflects the market’s choice of energy-efficient WGs and the promotion effectiveness of high EEG WGs. The EEG and RP data processing workflows are shown in Fig. 2: since both datasets (EEG information and WG sales) contain the “model” field, we first used the “query” syntax in SQL to fuse the two datasets and then sorted and summarized them based on the “month” and “province” fields to obtain the EEGs of different WG models in different provinces and months. Finally, the average EEG weighted by sales data was calculated. For instance, the value for IWMs can be calculated as follows:

$$\overline{EEG}=\frac{\sum \left({S}_{i}\times EE{G}_{i}\right)}{\sum {S}_{i}}$$

Here, $\overline{EEG}$ is the average EEG weighted by sales, *S*_*i*_ indicates the sales of IWM model *i*, and *EEG*_*i*_ indicates the EEG of IWM model *i*. Notably, as RP data are also included on the website mentioned above, we also calculated average RP weighted by sales data and provided the data in the CEEG dataset.

The ***economic/socioeconomic data*** used for predicting the regional average EEG weighted by sales in the REPM were retrieved from Wind (https://www.wind.com.cn/NewSite/edb.html) at the monthly level and were processed into the annual level for this study. This dataset contains macroeconomic and demographic indicators, including GDP per capita in constant prices, the average family size, the aging rate, the retail price index (RPI), the Engel coefficient, population, and living expenditures for consumption in purchasing household facilities.

#### Household data

To quantify the average EEG of each kind of WG at the household level, we developed a questionnaire survey and sent it to households in Beijing, China. The questionnaire was distributed to 21 randomly chosen subdistricts in the Haidian District (29 in total) in both 2020 and 2021. Systematic sampling was conducted in the chosen subdistricts with a random starting point in each subdistrict. Specially trained interviewers were responsible for interviewing the respondents. The participants were informed about the objectives, purposes and procedures of the survey at the beginning of the interview, and they signed an informed consent form that guaranteed the anonymity and confidentiality of the answers. During the survey, the participants were allowed to skip items and discontinue the questionnaire if they felt uncomfortable. After finishing the questionnaire, the participants were offered a small gift (worth about 10 RMB).

Economic/socioeconomic, demographic and age information, living and household factors, energy-use behaviors, the average EEG of each kind of WG in the household, and the attitude of HH towards high EEG WGs (EEG attitude) were collected from the questionnaire. The average EEG was recorded as a categorical variable, i.e., “high EEG (levels 1–2)” and “low to medium EEG (levels 3–5)”. A total of 1537 responses were collected, and 1327 of them were retained after cleaning and filtering. The items of the questionnaire used in developing the dataset and the prediction models can be found in the CEEG dataset.

### Data cleaning

For regional data, we mainly checked the uniqueness in the EEG information dataset and the matching of the EEG information and WG sales datasets. First, if one specific WG model is produced by more than one manufacturer, there will be multiple fields for the WG model in the EEG information dataset. The EEG information for such WG models was averaged and rounded upward to obtain the final result. Second, some WG models in the WG sales dataset were not listed in the EEG information dataset. To ensure the validity of the data, we retrieved information about these models on JD.com (https://www.jd.com), one of the largest online shopping website for HAs in China, and Zhongguancun Online (ZOL, http://www.zol.com.cn), a representative HA review portal. If the EEG information for the model was available on these websites, the missing EEG for the model in the EEG information dataset was completed; if not, the model information was removed from the WG sales dataset (details in Fig. S1 and Table S1).

To clean the household questionnaire data, a 3-step validation was conducted by (i) judging the validity of the questionnaire based on the completion level for each item; (ii) calculating energy consumption based on energy-use behaviors and comparing the value with the answer to the “total household energy use” item; and (iii) employing the 3-sigma principle to remove samples with outlier energy consumption values.

### Prediction methods

**Fig. 3:**
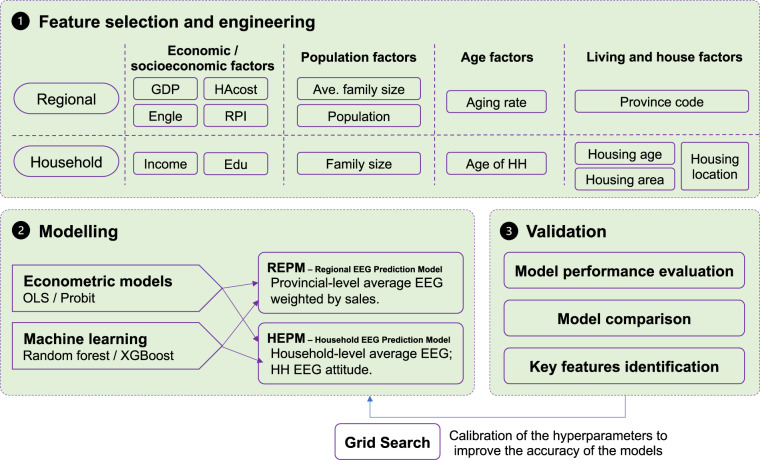
Workflow of the data-driven methods to predict the regional and household average EEG.

In addition to data acquisition and processing, an attempt was made to predict the average EEG of WGs at the regional and household levels based on various socioeconomic and household-related variables to explore future trends by establishing the REPM and HEPM. The workflow of the data-driven methods is shown in Fig. 3.

Since our household survey data might not be sufficient for time series predictions, we fit both the average EEG and the EEG attitude using household socioeconomic variables in the HEPM and attempted to use the latter to illustrate future trends in household average EEG.

#### Variable selection

For the REPM, all 8 indicators in the ***economic/socioeconomic data*** were selected as explanatory variables (or features in ML). The explained variables (or labels in ML) are the average EEG of a certain kind of WG. For the HEPM, the explanatory variables (or features in ML), including family income, family size, the age of the HH, and the educational level of the HH, are similar to those for the REPM. According to previous studies, living and housing factors (e.g., housing area) are found to be vital factors when predicting and explaining household energy use^19,20^. Thus, we added 3 living and housing variables (features), i.e., housing area, housing age, and housing location, in our HEPM to estimate their impacts on the average EEG in households and EEG attitude. Notably, variables such as the RPI are not considered in the HEPM, as they cannot be accurately decomposed to the household level based on our social survey data.

#### Modeling

According to the data characteristics (i.e., at the regional level, continuous variables; at the household level, categorical variables), traditional econometric methods (ordinary least squares (OLS) regression and probit classification) and ML algorithms (random forest (RF) and extreme gradient boosting (XGBoost)) were utilized. The effectiveness of traditional econometric methods and ML algorithms in predicting the average EEG was compared. The data were organized into two parts: 80% was randomly extracted for training, and 20% was randomly extracted for testing in the RF and XGBoost algorithms. To verify the ability of the methods to explain and predict the average EEG, we selected different indicators for different methods: for the REPM, which employs regression methods, the coefficient of determination (R^2^), root mean square error (RMSE), and mean absolute percentage error (MAPE) were calculated; for the HEPM, which deploys classification approaches, the precision, recall, and F1 score were calculated. To improve the fitness ability and accuracy of the methods, a grid search cross-validation procedure was employed.

The OLS and probit models were established using Stata 16.0, and the RF and XGBoost models were trained using Python 3.9. The meaning and descriptive statistics of the variables, the mathematical descriptions of the performance evaluation indicators, and the hyperparameters used in the methods and algorithms are all listed in Text S1, Tables S3, S4.

## Data Records

The CEEG dataset is publicly available in figshare^21^. The following data records can be accessed and downloaded in both *XLSX* and *JSON* formats: (1) the national and provincial market shares of the 5 kinds of WGs; (2) the national and provincial average EEGs and RPs of WGs weighted by sales; (3) household information, the average EEG and the EEG attitude; and (4) the variables and hyperparameters used in provincial- and household-level modeling. Additionally, the questionnaire both in Chinese and in English translation is provided. The files contained in the dataset are listed in Table 2.

**Table 2:** Summary of the dataset.

Level	Content	Document name
1. National level	**WG sales** *(Sorted by WG type and year; 2012*–*2019)*	1-1 Household appliance market share
	**EEG and RP weighted by sales** *(Sorted by WG type; 2012*–*2019 real sales data, 2020 predicted data)*	1-2 National – Weighted average EEG
2. Province level	**WG sales** *(Sorted by WG type and year; 2012-2019)*	2-1 Household appliance market share
	**EEG and RP weighted by sales** *(Sorted by WG type and province; 2012-2019 real EEG data, 2020 predicted data)*	2-2 Province – Impeller washing machine
		2-3 Province – Drum washing machine
		2-4 Province – Electrical water heater
		2-5 Province – Room air conditioner
		2-6 Province – Speed variable room air conditioner
3. Household level	**Average EEG of WGs** *(2019 & 2021, 1327 households in Beijing, China)*	3-1 Household basic information
		3-2 Average EEG and EEG attitude
4. Other information	**Variables and hyperparameters** of ML	4-1 Features (explanatory variables) in REPM
		4-2 Hyperparameters of the best models
	**Questionnaire**	4-3 Questionnaire in Chinese and English translations
	**Meta data**	Readme

For WG sales, the market shares of the studied WGs at both the national and provincial levels are provided, representing a total of 1240 data records. For weighted average EEG, 40 data records are included at the national level, and 1350 data records are included at the provincial level. For household information (including 8 variables), cleaned data records covering the 1327 valid household samples are uploaded to the dataset.

## Technical Validation

### Performance evaluation and comparison of different methods for average EEG prediction

The performance indicators for the REPM and HEPM are listed in Table 3. The R^2^ values of the best regression methods were all above 0.76, and the F1 scores of the best classification methods were all above 0.85, indicating that the models were generally well fitted. The ML algorithms performed better than traditional econometric methods at both the regional and household levels. In further analysis, we chose the methods that achieved the best performance for (i) each kind of WG in the REPM in terms of the RMSE or (ii) each kind of WG in the HEPM in terms of the F1 score. The XGBoost algorithm was more suitable for all methods.

**Table 3:** Model performance in predicting the provincial and household average EEG. Notes: Each ML algorithm were trained 100 times for each appliance. The reported model performance in the table, the computed 95% confidence interval (CI) in the subsequent text, and the predicted average EEG were based on the results of the 20 times with the lowest RMSE values.

Method	REPM						HEPM		
	Indicator	IWMs	DWMs	EWHs	ACs	VACs	Indicator	Average EEG	EEG attitude
Econometrics OLS/Probit	R^2^	0.24	0.77	0.41	0.52	0.72	F1	0.78	0.81
	RMSE	0.14	0.06	0.15	0.15	0.20	Precise	0.83	0.88
	MAPE	0.05	0.04	0.08	0.04	0.05	Recall	0.75	0.75
ML Random Forest	R^2^	0.73	0.76	0.86	0.68	0.88	F1	0.83	0.86
	RMSE	0.08	0.07	0.05	0.11	0.12	Precise	0.87	0.92
	MAPE	0.04	0.02	0.03	0.03	0.05	Recall	0.80	0.81
ML XGBoost	R^2^	0.78	0.90	0.89	0.76	0.93	F1	0.85	0.87
	RMSE	0.07	0.04	0.06	0.09	0.10	Precise	0.87	0.92
	MAPE	0.02	0.02	0.03	0.02	0.04	Recall	0.83	0.82

**Fig. 4:**
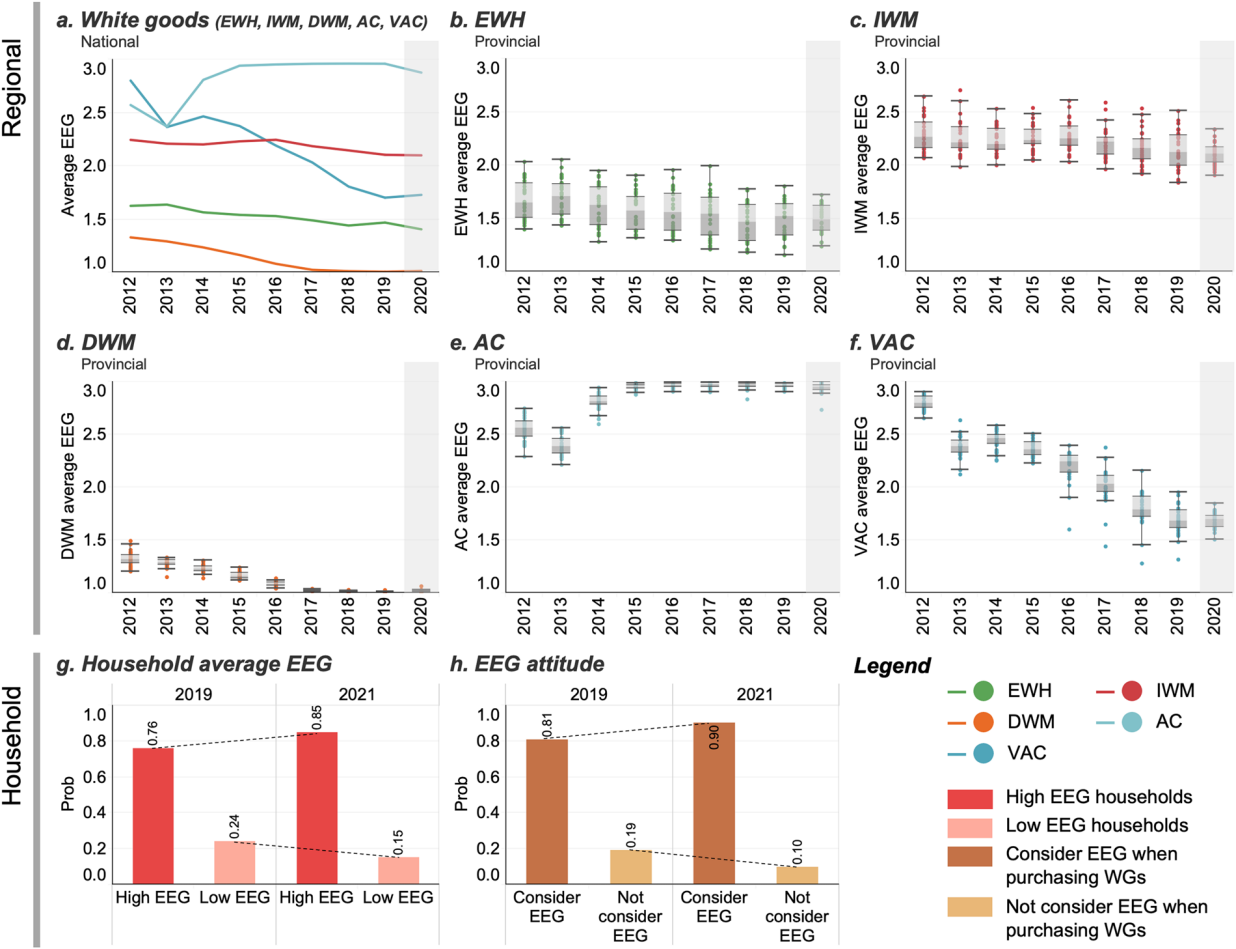
Average EEG at the regional and household levels. (**a**) National average EEG of the 5 kinds of WGs. The colors of the curves indicate the types of WGs. (**b**–**f**) Average EEG distribution in 30 provinces in mainland China. The shaded parts are predicted values calculated by the REPM, which will be analyzed in detail in the “Performance evaluation and comparison of different methods for average EEG prediction” section. (**g**) Distribution of the household average EEG. (**h**) Distribution of the EEG attitude.

For the predicted values (shaded part in Fig. 4), better performance, i.e., a higher average EEG, was predicted for IWMs, ACs and EWHs. For DWMs and VACs that had increasing market shares, the average EEG might decrease, i.e., be less energy efficient. These predicted values and their 95% confidence intervals (CIs) were also included in the dataset.

### Key factor identification

**Fig. 5:**
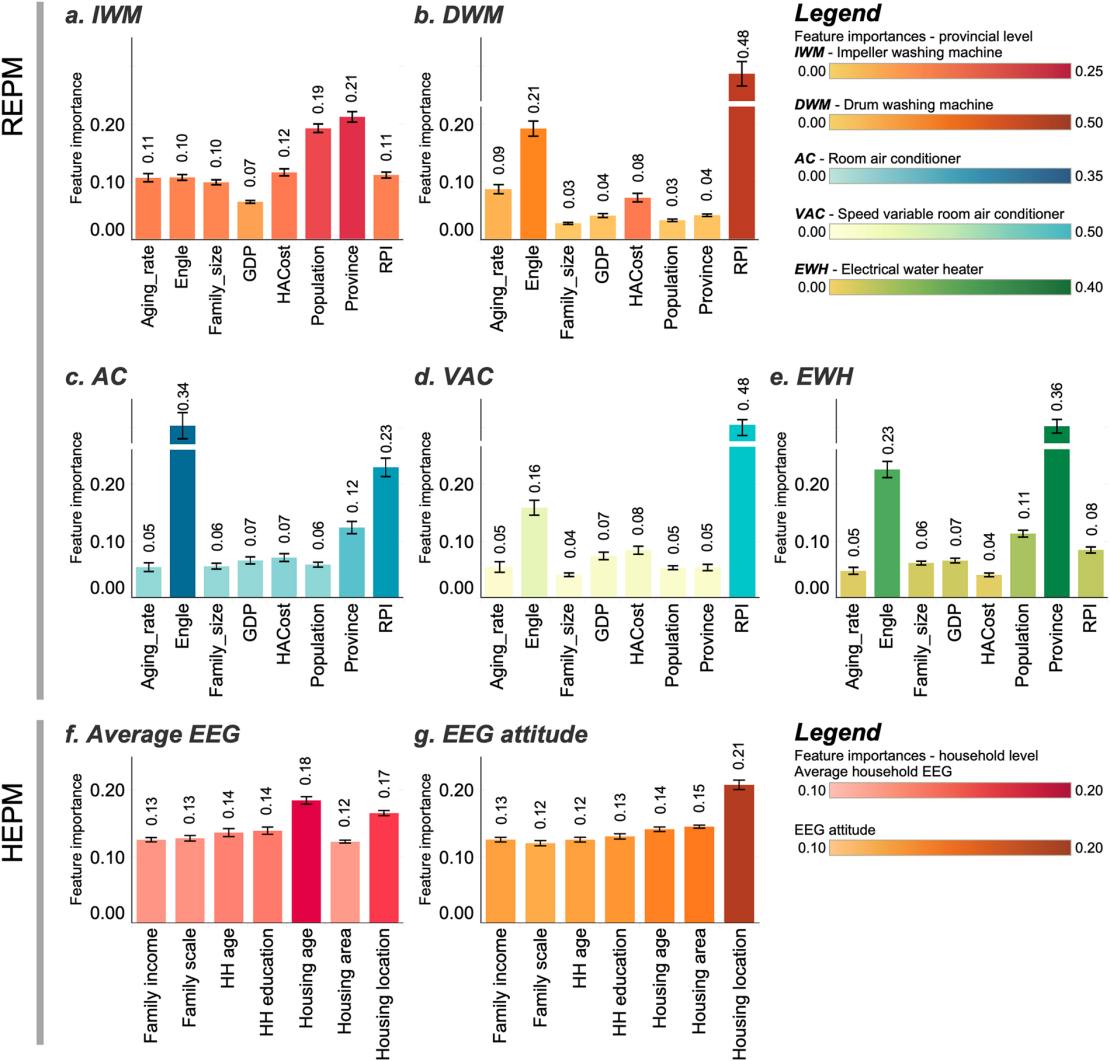
Importance of features in the REPM and HEPM. The shade of the color and the height of the bar indicate the importance of the features for each model. The error bars show the 95% CI of the feature importance.

For ML methods, the normalized importance of each feature can be calculated to reflect the level of importance in predicting the explained variable (i.e., in this study, the average EEG). Normalized importance (scaled from 0 to 1) refers to node impurity values for the Gini index (classification) or the mean square error (regression). The normalized importance values for each feature in the REPM and HEPM are available in Fig. 5.

We compared the importance of each feature and identified the features with the highest importance, which are referred to as the “key factors”. At the regional level, economic variables (especially the Engel coefficient and RPI) and the place variable are more important in predicting the EEG, i.e., they have a larger effect on the prediction results. For DWMs and VACs, the most important variable in predicting their average EEG is the RPI; for ACs, the Engel coefficient is the most important variable; and for EWHs, the place variable is the most important variable.

For the HEPM, housing variables, especially the housing location and housing age, are the most important variables in predicting both the average EEG and EEG attitude, which is similar to the fact that the place variable is the most important variable in the REPM for IWMs and EWHs. Economic/socioeconomic variables, including family income and HH education, also show significant impacts on the EEG, with importance of 0.27 and 0.26, respectively.

### Comparison with existing estimates

#### Regional data

To the best of our knowledge, there is no comparable provincial-level dataset for validation purposes. Therefore, in this section, we mainly discuss the methodology, the data trends at the national level and the prediction results.

The econometric methods and ML algorithms employed in this study have been widely used in the field of environmental studies, for instance, to predict household energy consumption^20,22^, to explain energy-use behaviors^23^, to analyze the impact of energy labels on HA sales^7,8^, to categorize the land or stream type^24^, etc.

Previous studies and data support our data trends at the national level from different perspectives:

- **For washing machines**, Haitong Securities stated in 2019 that the sales of IWMs and DWMs with a high EEG increased from 2012 to 2018 (https://bigdata-s3.wmcloud.com/researchreport/shift/a074be4ad1e3c479150201e30acf8336.pdf). A report released by the EU (https://www.switch-asia.eu/resource/analysis-of-the-current-situation-on-sustainable-consumption-in-china/) indicated that the sales of energy-saving washing machines (combining IWMs and DWMs) increased by 19.8% from 2016 to 2017 in China, which pushed the increase in the average EEG. These findings support our finding that the average EEG (weighted by sales of IWMs and DWMs) increased. ZOL stated in its white goods reports in 2019 and 2020 (URL: https://jd.zol.com.cn/738/7380835.html, https://jd.zol.com.cn/759/7591076.html) that the proportion of buyers’ preference for energy efficiency grade 1 (EEG 1) washing machines (mainly DWM models) decreased from 78% (2019) to 64% (2020), while a minimal change for the same period was observed for the proportion of EEG 2 washing machines, which are mainly IWM models. These conclusions are consistent with our predictions that the average EEG of DWMs would decrease slightly, while that of IWM would not change much (Fig. 4).
- **For water heaters**, as stated by Haitong Securities, the sales of energy-efficient water heaters (combining EWHs and gas water heaters) increased 6.5% from 2016 to 2017. This is in line with the increase in the average EEG weighted by sales of EWHs in our dataset.
- **For air conditioners**, the sales of high EEG ACs decreased, while that of VACs increased in the period from 2012 to 2018 according to Haitong Securities. Cheea.com and Gfk China also reported an increase in the sales of EEG 1 VACs based on their monitoring in the 2022 China Home Appliance Innovation Retail White Paper (http://upload.cheaa.com/2022/0712/1657611835227.pdf). These statements are aligned with the observed increase in the average EEG weighted by sales of VACs and the decrease in that of ACs.

#### Household data

For household-level datasets, we reviewed the literature^25,26^ for reference before designing and distributing the questionnaires. Similar EEG trends can be observed by analyzing data from the China Residential Energy Consumption Survey (CRECS). Taking the washing machine as an example, the percentage of households using a high EEG washing machine increased by approximately 10% from 2012 to 2014 in the CRECS data^27,28^, which is similar to our findings (i.e., the household average EEG increased by 9% from 2019 to 2021).

### Uncertainties, limitations and future work

#### Regional data

For regional data, the 95% CIs of the performance indicators of the XGBoost algorithm were calculated, with uncertainty ranges (defined as the lower and upper bounds of a 95% CI around the central estimates) of ±1.73%, ±3.08%, ±3.59% for the R^2^ value, RMSE and MAPE on average for the five kinds of WGs, respectively. The results show that there were relatively small uncertainties in the modeling process. We also provide the uncertainty range and the lower/upper limits of each predicted value in the CEEG dataset (files 2-2 Province – Impeller washing machine, 2-3 Province – Drum washing machine, 2-4 Province – Electrical water heater, 2-5 Province – Room air conditioner and 2-6 Province – Speed variable room air conditioner). The calculated uncertainty ranges for IWMs, DWMs, EWHs, ACs and VACs were ±0.71%, ±0.29%, ±0.72%, ±0.31% and ±1.02%, respectively, indicating a relatively small uncertainty in the prediction. However, the regional data still bear some inevitable uncertainties and limitations:

1. COVID-19 might involve some degree of uncertainty that would affect the predicted values. We trained the REPM with pre-COVID-19 data, and the prediction model performed well with acceptable values of the performance indicators. In addition, our trained average EEG trends were supported by the white goods reports by ZOL. However, the REPM data after COVID-19 were not trained because of the availability of WG sales data, and the model should be retrained with updated sales data from 2020 to the present in future work.
2. As a limitation, the raw online sales data that we used in this study cannot be disaggregated into provinces, as no further information about the sales destination was provided. Therefore, we used offline sales data as a weight when calculating the regional average EEG. However, online sales have become more dominant in China in recent years, and the proportion of online HA sales increased from less than 15% in 2012 to 69% in 2021 according to Statista (https://www.statista.com/statistics/1143294/china-share-of-household-appliance-online-sales/). The geographical distribution of online sales is likely to be different from that of offline sales. This difference may lead to bias in our study, and further efforts to obtain better refined online data are needed in future work.

With the public deployment of the CEEG dataset, the regional data will be updated annually if external data are released (e.g., annual socioeconomic statistics in China), as the predictions are based on these statistics. Additionally, with more detailed online sales information and water efficiency grade information available, a major version update covering both online and offline sales channels, revealing the impact of COVID-19 on the EEG and RPs, will be provided in future work.

#### Household data

For household data, to minimize potential uncertainties and/or errors, three steps were conducted before, during, and after the survey based on previous datasets^29–31^.

Prior to the survey, as a pilot study, the questionnaire was first distributed online to 15 researchers using convenience sampling. Based on their feedback, the wording of the items was improved, and the option settings for the family information items were adjusted (e.g., changing from collecting precise figures of family income to intervals of 50,000). Then, our sampling size was set to be *n* = 2000 based on the surveys in previous studies^8,9,25,26,32–37^ related to the EEG, household energy consumption, and energy-saving attitudes (Table S2 lists 10 representative studies with an average sample size of 675 and a maximum of 1082 at the provincial level).

To ensure the authenticity of the participants’ responses, interviewers with professional knowledge and extensive practical experience were invited to collect data through face-to-face interviews. A total of 1537 responses were collected, for a response rate of 76.9%.

After conducting the survey, we cleaned the data following the steps outlined in the Data cleaning section. Notably, the family income variable showed a high degree of dispersion (e.g., had an information entropy of 2.52), reflecting the inequality of the current household income state, which is consistent with other statistics (e.g., the GINI coefficient retrieved from Statista, URL: https://www.statista.com/statistics/250400/inequality-of-income-distribution-in-china-based-on-the-gini-index/), news reports (e.g., https://www.bloomberg.com/news/storythreads/2020-12-24/china-has-a-huge-wealth-gap-problem-and-it-s-getting-worse), and existing studies^38^.

In the modeling process, the F1 score, precision and recall had an average uncertainty range of ±1.04%, ±1.99%, ±1.01%, respectively, based on the use of cross-validation. We also evaluated the 95% CIs of the importance of each variable (including family income, family size, HH age, HH education, housing age, housing area and housing location). The average uncertainty ranges of the importance of the variables used to classify households with different average EEGs and EEG attitudes were ±3.07% and ±2.79%, respectively. These findings suggest that the HEPM modeling is robust.

To focus on the new developments of the household average EEG, updates will occur with each new round of questionnaire surveys in the case region (i.e., Beijing). In addition, if new rounds of surveys are conducted or extended to other regions, the model will be retrained, and the hyperparameters will be adjusted to account for different levels of development in each region.

## Usage Notes

The CEEG dataset, as listed in Table 2, can be directly utilized for various applications at the regional and household levels. The choice of *XLSX* and *JSON* formats enables accessibility for a wide range of data analysis tools. To help better understand the data, some extended analysis of the data, such as the market share and the trends of the average EEG weighted by sales, can be found in Fig. S2, Text S2–S4.

Some potential applications are proposed as follows. The regional data raise the possibility of identifying the driving factors behind average EEG changes, such as macroeconomic factors, demographic characteristics, and environmental indicators. Additionally, the average EEG and RP weighted by sales data can be included as vital information in the assessment system for evaluating the sustainability of provincial development, for instance, assessing the degree of green consumption. By exploring household data, researchers may gain a clearer understanding of how household factors influence both the average EEG and EEG attitudes toward the EEG. For instance, household data can be applied to compare the factors that affect EEG attitudes toward a high EEG among different household groups (e.g., households with a low average EEG *vs*. households with a high average EEG). Such attempts can provide crucial support for managing the EEG of WGs.

## Supplementary information

Supplementary Information

## Data Availability

Examples of the code that we used to produce the datasets presented in this paper (mainly for establishing REPM and HEPM) are provided in GitHub (https://github.com/CEEGDataset/CEEG-Dataset.git).
