# Supplementary material for: A dataset on energy efficiency grade of white goods in mainland China at regional and household levels
Source: Sci Data. 2023 Jul 12;10:445. doi: 10.1038/s41597-023-02358-x (PMC10338436; doi:10.1038/s41597-023-02358-x)
Supplement: Supplementary file 1 — Supplementary Information [file 41597_2023_2358_MOESM1_ESM.pdf]

Supplementary information for

A dataset on energy efficiency grade of white goods in mainland China at regional and household levels

Zonghan Li[1], Chunyan Wang[1]*, Yi Liu[1]
1 School of Environment, Tsinghua University, Beijing 100084, China
* Corresponding author: Chunyan Wang (wangchunyan@tsinghua.edu.cn)

Contents of this file:

7 SI Tables, 4 SI Texts, and 2 SI Figures

Figure S1 An example of RP information manual retrieval.
Figure S2 The market share of WG in the studied period.

Table S1 Details on obtaining EEG information by web crawling and manual retrieval.
Table S2 A review of existing surveys related to the EEG, household energy consumption, and energy-saving attitudes conducted in China.
Table S3 Descriptive statistics of the basic information in the household WG survey.
Table S4 The results of REPM OLS method.
Table S5 The results of HEPM Probit method.
Table S6 The optimized XGBoost model hyperparameters in REPM.
Table S7 The optimized XGBoost model hyperparameters in HEPM.

Text S1 Mathematical descriptions of the performance evaluation indicators.
Text S2 Explanations of three categories of HA.
Text S3 Explanations of the regional data.
Text S4 Explanations of the household data.

Data cleaning

Step 1: Search in China Energy Efficiency Label Website again.

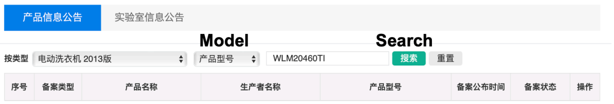

Step 2: Insert the model in the searching engine in ZOL.

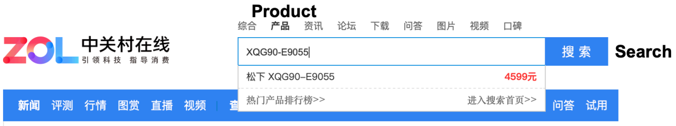

Step 3: Click the hyperlink of the search result to see more details.

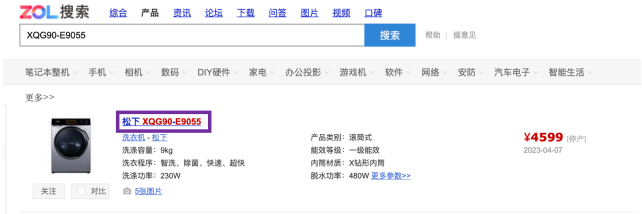

Step 4: Record the data provided by the website.

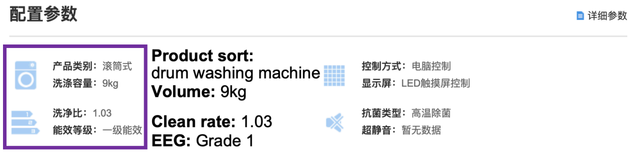

Figure S1 An example of RP information manual retrieval.

Table S1 Details on obtaining EEG information by web crawling and manual retrieval.

WGs	Models obtained by web crawling	Models obtained by manual retrieval	Sales percentage
IWM	7995	28	4.84%
DWM	3469	59	10.82%
EWH	4688	62	22.46%
AC	5673	282	46.29%
VAC	3987	608	34.17%

Note: Sales percentages are calculated by the total sales of models obtained by manual retrieval divided by that of models obtained by web crawling.

Descriptive statistics of basic information in household WG survey

Table S2 A review of existing surveys related to the EEG, household energy consumption, and energy-saving attitudes conducted in China.

Authors	Publication year (survey year)	Surveyed place	Samples	Population (unit: million)	EEG-related contents	Open to the public
CEEG (Household part)	(2019 & 2021)	Haidian District, Beijing, China	1327	3.13	Average EEG, EEG attitudes	Yes (after publication)
CGSS dataset[1]	2018 (2017)	China	1001 (Beijing) 12787 (China)	21.71 1390.08	EEG of one type of HA, HA use behaviours	Yes
CGSS dataset[2]	2015 (2014)	China	382 (Beijing) 10968 (China)	21.52 1367.82	EEG of four types of HAs, HA use behaviours	Yes
Zha et al.[3]	2017 (not mentioned)	China	325	1390.08	Attitude toward high-efficiency HAs	Yes
Wu, Zheng and Wei[4]	2017 (2014)	12 provinces in China	3404 (284 for each province on average)	709.88	Household energy consumption	Yes (Upon reasonable request)
Jiang et al.[5]	2022 (2020)	Guangzhou, Guangdong, China	1082	18.68	Household energy consumption	No
Wang et al.[6]	2011 (Not mentioned)	Beijing, China	816	20.19	Attitude toward saving electricity	No
Yu et al.[7]	2018 (2017)	Tongzhou District, Beijing, China	1017	1.50	HA use behaviours	No
Wang et al.[8]	2020 (Not mentioned)	Jilin, China	835	24.07	Willingness to pay for high EEG HAs	No
Zhang et al.[9]	2021 (2018)	Beijing, China	1003	21.54	Attitude and willingness to pay for high EEG HAs	No
Jiang et al.[10]	2016 (Not mentioned)	Tianjin, China	504	5.62	HA use behaviours	No

Table S3 Descriptive statistics of the basic information in the household WG survey.

Variable	Description	Type	Mean (SD) or Median (IE)	Min-Max
Family size	Household size, persons	Continuous	2.92 (1.04)	1-6
Housing location	Live within which ring road?	Continuous	4.40 (1.00)	3-6
Housing area	Housing area (including common share)	Continuous	69.30 (18.82)	12-190
HH edu	Educational level of the head of household<br>*Uneducated or primary=1, Secondary=2, Undergraduate=3, Postgraduate or higher=4*	Categorical	3 (1.82)	1-4
HH age	Age of the head of household<br>*<18=1, [18,30]=2, [30,40]=3, [40,50]=4, [50,60]=5, >60=6.*	Categorical	4 (2.20)	2-6
Family income	Household income, 10 thousand yuan<br>*[0,5]=1, (5,10]=2, (10,15]=3, (15,20]=4, (20,25]=5, (25,30]=6, (30,$+\infty$)=7, unknow or blank=0*	Categorical	3 (2.52)	0-7
Housing age	In which year was the housing you live in built?<br>*<1980=1, 1980s=2, 1990s=3, [2000-2005]=4, (2005-2010]=5, (2010-2015]=6*	Categorical	3 (1.87)	1-5

Notes: For continuous variables, the mean and standard deviation values were calculated; for categorical variables, the median and information entropy were calculated.

Econometric model establishment

Table S4 The results of REPM OLS method.

Variables	VIF	IWM	DWM	EWH	IC	AC	VAC
GDP per capita	3.53				–		+
RPI	1.49		–	–	+	+	–
Population	1.21		+	+			+
Aging rate	1.83	–	–			+	–
Engel coefficient	1.52	–	+	+	+	–	+
Family scale	2.30	–	–	+	–	+	–
HACost	3.46	–	–		+		–

Note: Blank values for the variables in the table indicates that the variables are not significant in the model; values of '+' indicate that the variables are significant and the coefficients are positive; values of '-' indicate that the variables are significant and the coefficients are negative. Smaller values of the explanatory variables indicate more energy efficiency. The VIF values in the table measure the multicollinearity of the model. According to Chen[11], a model is considered to have no strong multicollinearity when VIF is less than 10, and the VIF values of the models all meet this condition.

Table S5 The results of HEPM Probit method.

Variables	General EEG (binary)	EEG attitude (binary)
HH education		
HH age	–	
Family income	+	
Family scale		–
House area		+
House location	–	
House era	–	

Note: Blank values for the variables in the table indicates that the variables are not significant in the model; values of '+' indicate that the variables are significant and the coefficients are positive; values of '-' indicate that the variables are significant and the coefficients are negative.

Machine learning model establishment

Table S6 The optimized XGBoost model hyperparameters in REPM.

XGBoost Regional average EEG	IWM	DWM	EWH	AC	VAC
learning_rate	0.03	0.05	0.05	0.1	0.1
max_depth	8	7	4	8	3
min_child_weight	1	1	3	2	1
gamma	0.0	0.0	0.0	0.0	0.0
subsample	0.75	0.75	0.85	0.85	0.85
colsample_bytree	0.75	0.75	0.75	0.75	0.75
objective	reg:gamma	reg:gamma	reg:gamma	reg:gamma	reg:gamma
nthread	4	4	4	4	4

Note: The hyperparameters that critically influence the model performance are listed in the table, while other hyperparameters which have little or no influence on the model performance follow the default values.

Table S7 The optimized XGBoost model hyperparameters in HEPM.

XGBoost Provincial EEG	Average EEG	EEG attitude
learning_rate	0.03	0.03
max_depth	3	3
min_child_weight	2	2
gamma	0.3	0.3
subsample	0.85	0.85
colsample_bytree	0.75	0.75
objective	binary: logistic	binary: logistic
nthread	4	4

Note: The hyperparameters that critically influence the model performance are listed in the table, while other hyperparameters which have little or no influence on the model performance follow the default values.

Text S1 Mathematical descriptions of the performance evaluation indicators.

$$Precision = \frac{TP}{TP+FN} \quad (1)$$

$$Recall = \frac{TP}{TP+FP} \quad (2)$$

$$F1\_score = \frac{2\times(Precision \times recall)}{Precision + recall} \quad (3)$$

$$R^2 = 1 - \frac{\sum_{i=1}^{n}(\hat{Y}_i - Y_i)^2}{\sum_{i=1}^{n}(\bar{Y} - Y_i)^2} \tag{4}$$

$$RMSE = \sqrt{\frac{\sum_{i=1}^{n}(\hat{Y}_i - Y_i)^2}{n}} \tag{5}$$

$$MAPE = \frac{\sum_{i=1}^{n}\left|\frac{(\hat{Y}_i - Y_i)}{Y_i}\right|}{n} \tag{6}$$

Note: In equations (1) and (2), TP means the actual category is 1, and the identification category is 1; FN means the actual category is 1, and the identification category is 0; FP means the actual category is 0, and the identification category is 1; TN means the actual category is 0, and the identification category is 0.

Additional explanations on regional data

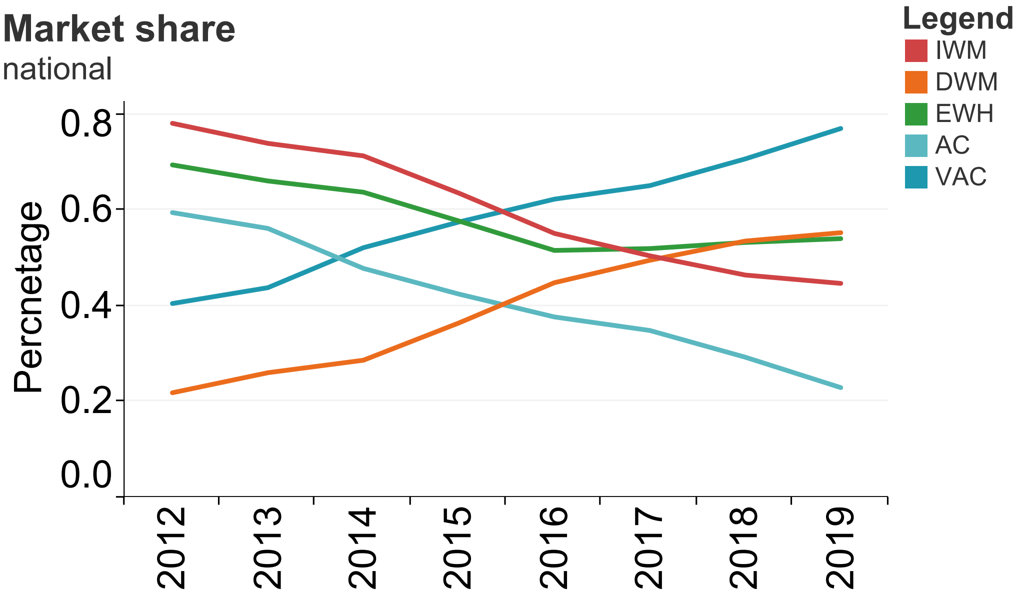

Figure S2 The market share of WG in the studied period.

The colors of the curves indicate the types of WG. The market share here means the proportion of sales of one certain kind of WG to the total sales of WG that can perform similar functions. For instance, the market share of AC equals the proportion of AC sales to AC and VAC sales. In the calculation, IWM and DWM are considered to constitute the washing machine market, EWH and GWH to constitute the water heater market, and AC and VAC to constitute the air conditioner market.

Text S2 Explanations of three categories of WGs.

To facilitate discussion, the studied appliances are divided into 3 categories based on market performance (**Figure S2**), the relationship with socioeconomic variables, and other factors: the phase-out category (IWMs, ACs), phase-in category (DWMs, VACs, DWs), and compete-with-gas category (EWHs). For the phase-out category, ACs were completely withdrawn from the market in 2021 due to the revision of EEG-related standards, and their market share declined during the studied period; IWMs might be gradually replaced by DWMs due to their disadvantages, such as more severe damage to clothes and higher water consumption. For the phase-in category, DWMs and VACs replace the original market shares of IWMs and ACs, respectively. For the compete-with-gas category, EWHs have gas water heaters that perform similar functions but use gas to compete with them.

Text S3 Explanations of the regional data.

From 2012 to 2019, the market share of WGs in the phase-out and compete-with-gas categories decreased. Specifically, the market share of IWMs decreased from 78% to 45%, that of ACs decreased from 59% to 23%, and that of EWHs decreased from 69% to 54%. In contrast, the market share of WGs in the phase-in category

increased. Specifically, the market share of DWMs increased from 22% to 55%, and that of VACs increased from 41% to 77%.

As shown in **Fig. 4a** in the main text, the energy efficiency of IWMs, DWMs, EWHs, and VACs in the 2012-2019 period showed upward trends (i.e., the EEG values decreased). This result indicates that WGs purchased in China were more energy efficient. However, the opposite trend was observed for ACs. Additionally, a significant shock to the average EEG of ACs was observed in 2014. This finding might be because of the end of energy-efficient household appliance subsidy policies in 2013, according to All View Cloud (https://www.avc-mr.com/article/detail?id=228166870681780224). Thus, consumers tended to buy cheaper but less energy-efficient ACs. At the provincial level, the trends of the upper pivot, median, and lower pivot of the box line plot (**Fig. 4b** to **Fig. 4f** in the main text) of the provincial average EEG distribution over time were all similar to the trends of the national average EEG.

Text S4 Explanations of the household data

According to the survey data, the existing WGs in 79% of households were grades 1-2 (i.e., high EEG), and when purchasing WGs, 87% of households considered the EEG to be a vital factor. That is, in most of the surveyed households, behaviors and preferences with regard to purchasing and using WGs tended toward energy-saving WGs. Additionally, **Fig. 4g** and **Fig. 4f** show that from 2019 to 2021, the percentage of high average EEG households increased from 76% to 85%, and the percentage of households considering the EEG when purchasing WGs grew from 81% to 90%. Therefore, residents' attitudes toward acquiring energy-efficient WGs are becoming more positive, and households are gradually becoming energy-efficient in the process of gradually updating their WGs. This indicates that more households could have a higher average EEG in the future.

References

1 Renmin University of China. Chinese General Social Survey 2018. Chinese National Survey Data Archive. http://www.cnsda.org/index.php?r=projects/view&id=35694191 (2018).

2 Renmin University of China. *Chinese General Social Survey 2015*. Chinese National Survey Data Archive. http://www.cnsda.org/index.php?r=projects/view&id=62072446 (2015).

3 Zha, D., Yang, G., Wang, W., Wang, Q. & Zhou, D. Appliance energy labels and consumer heterogeneity: A latent class approach based on a discrete choice experiment in China. *Energy Econ.* **90**, 104839 (2020).

4 Wu, S., Zheng, X. & Wei, C. Measurement of inequality using household energy consumption data in rural China. *Nat. Energy* **2**, 795-803 (2017).

5 Jiang, L., Shi, X., Wu, S., Ding, B. & Chen, Y. What factors affect household energy consumption in mega-cities? A case study of Guangzhou, China. *J. Clean Prod.* **363**, 132388 (2022).

6 Wang, Z., Zhang, B., Yin, J. & Zhang, Y. Determinants and policy implications for household electricity-saving behaviour: Evidence from Beijing, China. *Energy Policy* **39**, 3550-3557 (2011).

7 Yu, M., Wang, C., Liu, Y., Olsson, G. & Bai, H. Water and related electrical energy use in urban households—Influence of individual attributes in Beijing, China. *Resour. Conserv. Recycl.* **130**, 190-199 (2018).

8 Wang, X. e. et al. Urban consumers' willingness to pay for higher-level energy-saving appliances: Focusing on a less developed region. *Resour. Conserv. Recycl.* **157**, 104760 (2020).

9 Zhang, Y. & Tao, W. Will energy efficiency affect appliance price? An empirical analysis of refrigerators in China based on hedonic price model. *Energy Policy* **147**, 111818 (2020).

10 Jiang, S. et al. Residential water and energy nexus for conservation and management: A case study of Tianjin. *Int. J. Hydrog. Energy* **41**, 15919-15929 (2016).

11 Chen, Q. Advanced Econometrics and Stata Applications. 2nd edn. (Higher Education Press, 2014).
